# Association between career adaptability and turnover intention among nursing assistants: the mediating role of psychological capital

**DOI:** 10.1186/s12912-023-01187-y

**Published:** 2023-02-02

**Authors:** Changxian Sun, Yurong Xing, Yuting Wen, Xia Wan, Yaping Ding, Yan Cui, Wenhui Xu, Xiaoxiao Wang, Hongling Xia, Qian Zhang, Min Yuan

**Affiliations:** 1 Nanjing Medical University, Nanjing, China; 2 Jiangsu Vocational Institute of Commerce, Nanjing, China; 3 The Affiliated Wuxi People’s Hospital of Nanjing Medical University, Wuxi, China; 4 Jinshen College of Nanjing Audit University, Nanjing, China

**Keywords:** Nursing assistant, Long-term care, Turnover intention, Career adaptability, Psychological capital

## Abstract

**Background:**

High turnover intention of nursing assistants was detrimental to the sustainability of long-term care. Career adaptability is an important determinant in reducing turnover intention, but little research has explored the mechanism from the perspective of psychological capital. The aim of this study was to analyze the association between career adaptability and turnover intention and to examine the mediating role of psychological capital between career adaptability and turnover intention among nursing assistants in mainland China.

**Methods:**

A cross-sectional online study was conducted among 276 nursing assistants from eight nursing homes in Nanjing, China. The participants’ career adaptability, psychological capital, and turnover intention were obtained. SPSS 26.0 and Amos 24.0 software were employed for statistical analysis.

**Results:**

Career adaptability was positively related to psychological capital and negatively linked to turnover intention (*P* < 0.01). Psychological capital played a fully mediating role (*β* = -0.085, *P* < 0.05) in the relationship between career adaptability and turnover intention, and the largest indirect effect was generated through the curiosity dimension.

**Conclusions:**

The management of long-term care facilities should focus on assessing the level of career adaptability of nursing assistants. The overall improvement of career adaptability and psychological capital is conducive in reducing turnover intention. Targeted interventions are recommended to improve career adaptability and reduce turnover intentions by increasing career curiosity. Online career adaptability programs can be developed for nursing assistant students to improve their psychological capital and facilitate career transitions.

## Background

China has the largest population of older adults worldwide and the number is surging rapidly. The statistics of the seventh census showed that older adults aged over 60 years old accounted for 18.9% of the total population [1]. Those over 65 years old were 200.56 million by the end of 2021 in China and it is estimated to reach 300 million by 2035 [2]; this requires large amounts of care for the older people. Traditionally, the aged were supported by home-based care. Nevertheless, with the small family structure and urbanization, the traditional home-based care has been undermined and the demands for professional long-term care (LTC) are increasing nowadays [3]. Nursing assistants (NAs) were relied upon to provide the majority of hands-on care for residents and offer interpersonal interaction with them in LTC facilities [4].

In recent years, recruiting and retaining the workforce of NAs have long been critical challenges [5]. In the US, the annualized turnover rate of NAs was the highest at 74.5% among all the employees in care homes [6]. In 2020, there were only 0.3 million NAs in China, and their turnover rate was reported to be between 40 and 70%, while that of NAs students in their first year was about 30% after graduation [7], which was detrimental to the sustainability of LTC workforce.

Turnover intention is used to measure employees’ intention to resign from their current organization and look for another job [8]. It is confirmed to be the most immediate determinant of actual turnover behaviors and turnover rates [9]. A study conducted in 162 Swiss nursing homes showed that 56% of NAs reported the intention to leave [10]. In the US, 45% NAs reported that they were very likely or somewhat likely to leave their current job in the next year [6]. Li surveyed 1,390 NAs and found that 51.8% had turnover intention [11]. NAs are required to take on physically demanding work, receive limited training, have low wages, and own limited prospects in terms of their career advancements [5]. The majority of Chinese NAs are women aged 40–60 years with a diploma below middle school [12], and they usually need to work either 8/12 h or 24-h shifts [13]. Most of them are transited from landless peasants or industrial workers [14]. It has been shown that salaries, work benefits [15], and job satisfaction[16] are negatively associated with turnover intention. A study revealed that a positive organizational culture also helped to retain employees [17]. In addition, NAs who expose to job burnout [18] and psychosocial stress [10, 19] tend to have high intention to leave. For residents, high levels of turnover intention result in nursing disruption and poor health outcomes, such as pain, urinary tract infections and more readmissions [20–23]. For organizations, it can be costly in terms of recruiting and training new employees [24]. Therefore, understanding the factors influencing caregivers' turnover intention and improving them has become an important issue to be addressed in the development of the global aging business.

Career adaptability is described as the readiness to deal with predictable and unpredictable problems caused by work tasks, occupational transitions and work traumas [25, 26]. Savickas defined the operational concept of it in four aspects: concern, control, curiosity, and confidence [26]. As the result of interaction between individuals and environment, career adaptability enables individuals to adapt well to the challenges in work environment and have a positive attitude towards future career development [27]. Promotability exhibited positive correlations with career adaptability as well [17]. Employees with high career adaptability have higher work engagement and well-being [28]. Conversely, employees who lack career adaptability cannot successfully cope with the work tasks, and they will feel less job satisfaction and be inclined to leave the organization[16]. Current research on career adaptability and turnover intention has focused on enterprise employees [16, 27, 29], while little research has been conducted on NAs in LTC facilities. According to traditional Chinese beliefs, NAs are considered undervalued jobs because employees are not obliged to possess professional certifications to enter this position [30]. Highly educated NAs students are might unable to adapt to the work conditions after graduation or even consider quitting due to a lack of self-worth and social identity [31]. The ‘14th Five-Year Plan’ issued by the State Council in 2021 underlined the necessity of expanding the number of NAs and establishing aged care majors in higher education institutions [32]. In the context of an aging population, clarifying the specific mechanism between career adaptability and turnover intention helps to promote quantity and quality of the aged care workforce.

Previous studies have shown that career adaptability is a crucial factor in predicting turnover intention [17, 29]. Current research has focused on the relationship between them in the view of organizational environment [33](e.g. LTC setting) and job-related factors, such as work social support [29], promotability and job satisfaction [17]. According to Career construction theory [25], individuals with higher career adaptability own more psychosocial resources that enable them to successfully adapt and handle career tasks. In addition, turnover intention can be influenced by internal psychological factors because it is a subjective individual tendency to make career decisions. Thus, exploring the potential psychological mediators between career adaptability and turnover intention is valuable to further understanding this relationship.

As the center of psychological resources, psychological capital is a positive mental capacity that individuals generate in active emotional states for personal growth and organizational constructions [34]. Employees who possess high levels of psychological capital are confident about their abilities to successfully carry out the action plans (self-efficacy); committed to pursuing meaningful goals and finding alternative methods to achieve success (hope); able to overcome and recover from setbacks (resilience); and inclined to hold a positive view by attributing events to favorable aspects (optimism) [35]. Evidence has been presented that psychological capital has a positive impact on employees' attitudes towards work, including job satisfaction and organizational commitment [36]. Earlier studies have found that career adaptability has a positive impact on hope, resilience, and self-efficacy [37–39], all of which are major components of psychological capital. In addition, it has been found that psychological capital can contribute to reducing burnout and enhancing career identity [40], and it has a positive effect on reducing turnover intention [18, 41]. In addition, Yim [42] found that psychological capital could mediate the association between occupational stress and turnover intention. Therefore, psychological capital can be seen as a potential mediator between career adaptability and turnover intention, contributing to further understanding the relationship from a psychological perspective and developing more precise intervention strategies for NAs.

**Fig. 1:**
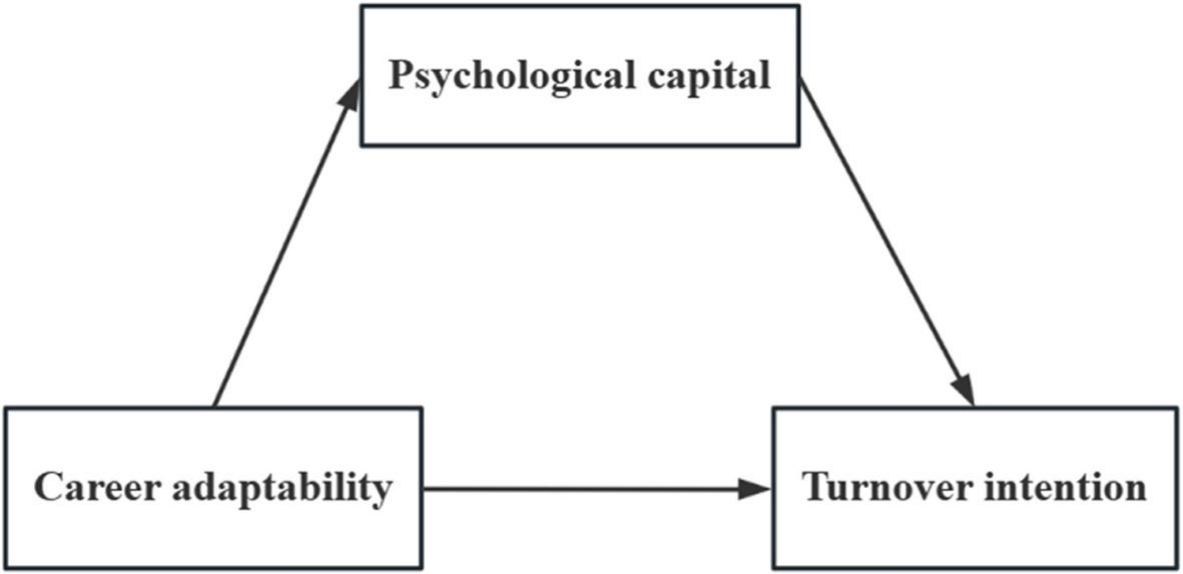
The theoretical mediation model

This study aimed to investigate the relationship between career adaptability and turnover intention, and determined whether psychological capital is a potential mediating factor in the relationship. Based on these, we proposed the following hypotheses: 1) Career adaptability will be negatively related to turnover intention; 2) Career adaptability will be positively related to psychological capital; 3) Psychological capital will mediate the relationship between career adaptability and turnover intention. Figure 1 presented the conceptual framework.

## Methods

### Ethical considerations

The research was approved by the Ethics Committee of the Nanjing Medical University (NO.2021(605)), and adhered to the Declaration of Helsinki. All eligible participants were informed of the study aims and methods. Their participation was voluntary, and they had the right to withdraw from the survey at any time. The informed consent was obtained from all study participants.

### Settings and samples

A convenient sampling method was used in this cross-sectional study. The participants were recruited from eight nursing homes in Nanjing, Jiangsu province, China. These nursing homes are located in urban and rural areas. The inclusion criteria were as follows: (1) aged over 18 years old; (2) have been employed in nursing homes for at least one month; (3) directly involved in hands-on care of residents. The exclusion criteria were: (1) having difficulty filling out the questionnaires; (2) failing to provide direct care for residents. According to the recommendation of Kline [43], a sample size of more than 200 is desirable for Structural Equation Modeling (SEM), at which point a failure of the chi-square test may indicate that the problem is severe enough to reject the model. If the sample size is less than 200, it is difficult to provide sufficient statistical power and tenable conclusions. A total of 276 participants completed the questionnaires.

### Data collection

Due to the requirements of COVID-19 control, data collection was conducted on a Chinese free online platform (https://www.wjx.cn/) from June 2021 to January 2022. Two college students majoring in service and management received standardized training. A 3-day pre-survey was conducted before the start of the study to ensure that investigators were qualified. The study’s purposes were explained to the managers of nursing homes and obtained their support. A link and Quick Response (QR) code were distributed to participants to fill out the questionnaire. The information about this study and informed consent forms were sent to eligible participants via social media. When participants clicked on the link or scanned the QR code to access the questionnaire website, they could see the contents of the informed consent form on the first page of the questionnaire. To avoid the situation that the same participant completed multiple questionnaires, the account and IP address were set to have only one chance to submit. It was estimated that participants would take 5 to 10 min to complete the questionnaire. Submission of a completed questionnaire was considered as voluntary participation in this study.

### Measurement

A structured questionnaire was used to collect data including the following four subsections: Demographic questionnaire, Career Adapt-Abilities Scale (CAAS), Psychological Capital Scale (PCS) and Turnover Intention Scale (TIS). ∙

#### Demographic questionnaire

The demographic characteristics included gender, age, years of work, educational level, marital status, residence, monthly income, the number of received nursing training, professional qualification certificate, family support, the number of residents cared for per day, and the extent to which knowledge meets aged care needs.

#### The Chinese version of the career adapt-abilities scale (CAAS)

The CAAS was used to assess career adaptability. The scale was developed by Savickas et al. [26] and translated by Hou [44]. The 24 items in the scale are divided into four dimensions: concern, control, curiosity, and confidence. Each item is scored on a 5-point Likert scale (ranging from 1 = *completely inconsistent* to 5 = *completely consistent*). The total scores range from 24 to 120, with a higher score representing better career adaptability. This scale has been tested in different cultural contexts which has an excellent reliability and the Cronbach’s α coefficient of the Chinese version in this study was 0.964. The split-half reliability was 0.921. The Cronbach’s α coefficient for each dimension were 0.864, 0.932, 0.901and 0.939.

#### The Chinese version of the psychological capital scale (PCS)∙

The PCS was designed by Luthans et al. [34] for measuring staff’s psychological capital. The scale was translated and revised by Li [45]. It consists of 24 items scored on a 6-point Likert scale (ranging from 1 = *strongly disagree* to 6 = *strongly agree*), but three items are reverse scores. This scale is divided into four dimensions: self-efficacy, hope, resilience and optimism. The total scores range from 24 to 144, with higher scores indicating higher levels of psychological capital. PCS has been confirmed to have high reliability and validity in China [18], and the Cronbach’s α coefficient was 0.928 in this study. The split-half reliability was 0.833. The Cronbach’s α coefficient for each dimension were 0.861, 0.878, 0.756 and 0.667.

#### The Chinese version of the Turnover Intention Scale (TIS)∙

The TIS was used to measure employees’ willingness to leave their current organization. The scale was developed by Farh [46] in1998 and was widely used in China. It has four items scored on a 5-point Likert scale (ranging from 1 = *completely inconsistent* to 5 = *completely consistent*). Among them, item three is the opposite scoring method. The total scores range from 4 to 20, with higher scores indicating more turnover intention. The Cronbach’s α coefficient of the scale was 0.828 in this study and the split-half reliability was 0.758.

### Data analysis

SPSS 26.0 was used for statistical analysis. Categorical variables (such as gender, residence, etc.) were described by frequency and percentage, and continuous variables conforming to a normal distribution were described by means and standard deviation (M ± SD). The univariate analysis was examined using an independent *t*-test and one-way analysis of variance (ANOVA). When the difference between groups was significant, a post hoc test for multiple comparisons was conducted using Scheffé's test. Pearson correlation analysis was performed to explore the relationship between career adaptability, psychological capital and turnover intention. Multiple linear regression was used to analyze the association between career adaptability and turnover intention after adjusting for demographic factors. To decide which independent variables to include in the multiple regression, categorical variables were used for implementation: years of work (≤ 1; > 1 and ≤ 3; > 3 and ≤ 5; > 5 and ≤ 10; > 10)).

Amos 24.0 software was performed to test the mediation effects. The 5000 bootstrapped samples method with 95% bias-corrected confidence interval (CI) estimation was used to assess whether psychological capital mediated the relationship between career adaptability and turnover intention. The proportion of mediating effect was calculated as the indirect effect divided by the total effect(a*b/c). A value of *P* < 0.05 was considered statistically significant.

## Results

### Demographic characteristics of participants

Among the 280 eligible participants, 276 respondents completed the total questionnaires, resulting in an effective response rate of 98.6%. The demographic characteristics of participants are shown in Table 1. The age of most participants was between 40 and 49 (68.8%), and there were more females (86.2%) than males (13.8%). 66.7% worked for less than five years and 17.0% worked for more than ten years. Regarding the level of education, 59.1% had a middle school education or less. 63.0% have a monthly income of less than RMB 4,000 ($597.60).

**Table 1:** Demographic characteristics of participants and the turnover intention(*N* = 276)

Variables	N (%)	Turnover intention	*t/F*	*p*
Gender				
Male	38(13.8)	10.83 ± 3.35	0.677	0.499
Female	238(86.2)	10.47 ± 3.01		
Age(year)				
< 30	44(15.9)	10.64 ± 2.62	0.123	0.946
30–39	40(14.5)	10.43 ± 2.72		
40–49	108(39.1)	10.41 ± 2.97		
≥ 50	84(30.4)	10.64 ± 3.53		
Years of work				
≤ 1 ^a^	107(38.8)	10.29 ± 2.90	4.062	0.003
> 1 and ≤ 3 ^b^	56(20.3)	10.61 ± 3.08		
> 3 and ≤ 5 ^c^	21(7.6)	11.35 ± 1.69		
> 5 and ≤ 10 ^d^	45(16.3)	11.74 ± 3.42		
> 10 ^e^	47(17.0)	9.41 ± 3.09		
		d > e		
Educational level				
Elementary school	33(12.0)	10.79 ± 3.67	0.102	0.959
Middle school	130(47.1)	10.46 ± 2.99		
High school	47(17.0)	10.52 ± 3.30		
College and above	66(23.9)	10.51 ± 2.71		
Marital status				
Unmarried	45(16.3)	10.89 ± 2.54	1.177	0.319
Married	227(82.2)	10.41 ± 3.15		
Divorced	3(1.1)	13.33 ± 1.53		
Widowed	1(0.4)	11.00 ± 0.00		
Residence				
Countryside	196(71.0)	10.73 ± 2.98	1.799	0.073
City	80(29.0)	10.00 ± 3.20		
Monthly income (RMB)				
≤ 3000	61(22.1)	10.32 ± 2.96	3.775	0.011
3001–4000	113(40.9)	10.76 ± 2.91		
4001–5000	67(24.3)	11.06 ± 3.07		
> 5000	35(12.7)	9.06 ± 3.31		
		d < b, c		
The number of nursing trainings received received				
≤ 2	142(51.4)	10.87 ± 3.10	1.952	0.052
> 2	134(48.6)	10.15 ± 2.98		
Professional qualification certificate				
Yes	137(49.6)	10.96 ± 2.99	-2.410	0.017
No	139(50.4)	10.08 ± 3.07		
Family support				
Yes	244(88.4)	10.40 ± 3.09	1.812	0.165
No	7(2.5)	12.14 ± 2.34		
Uncertain	25(9.1)	11.21 ± 2.70		
The number of residents cared for per day				
≤ 10	212(76.8)	10.34 ± 3.20	-2.120	0.036
> 10	64(23.2)	11.13 ± 2.43		
The extent to which knowledge meets aged care needs				
Absolutely	53(19.2)	10.75 ± 3.84	1.276	0.280
Mostly	164(59.4)	10.70 ± 2.80		
Partly	45(16.3)	10.01 ± 2.92		
Not at all	12(4.3)	9.00 ± 3.02		
Unknown	2(0.7)	10.50 ± 0.71		

*TIS* Turnover intention scale

### Differences in variables based on demographic characteristics

There were significant differences in turnover intention based on years of work and monthly income. Specifically, participants who have worked for more than 5 years but less than 10 years had higher scores of turnover intention than those who worked for over 10 years (*F* = 4.062, *P* = 0.003). Participants with monthly income of more than RMB 5,000 had lower scores of turnover intention than the other two groups (*F* = 3.775, *P* = 0.011). In addition, participants who owned professional qualification certificate scored significantly higher than those who without (*t* = -2.410, *P* = 0.017). Participants who cared for less than ten residents per day was significantly lower than those who cared for over ten residents (*t* = -2.120, *P* = 0.036). Table 1

### Description of career adaptability, psychological capital, and turnover intention

Table 2 showed descriptions of career adaptability, psychological capital and turnover intention. The total mean score of turnover intention was 10.52 ± 3.06. The total mean score of career adaptability was 96.87 ± 15.38; and the mean scores of the dimensions “concern” “control” “curiosity” and “confidence” were 22.96 ± 4.73, 24.70 ± 4.22, 24.19 ± 4.11, and 25.03 ± 4.18, respectively. The total mean score of psychological capital was 108.78 ± 13.09.

**Table 2:** Descriptive statistics and Pearson correlation analysis results of study variable (*N* = 276)

Variable	Mean	SD	1	2	3	4	5	6	7
1. TIS	10.52	3.06	1						
2. CAAS	96.87	15.38	-0.176^**^	1					
3. Concern	22.96	4.73	-0.110	0.839**	1				
4. Control	24.70	4.22	-0.183**	0.913**	0.653**	1			
5. Curiosity	24.19	4.11	-0.144*	0.915**	0.675**	0.801**	1		
6. Confidence	25.03	4.18	-0.194**	0.907**	0.631**	0.820**	0.808**	1	
7. PCS	108.78	13.09	-0.203**	0.556**	0.414**	0.530**	0.507**	0.541**	1

*TIS* Turnover intention scale, *CAAS* Career adapt-abilities scale, *PCS* Psychological capital scale

^*^P < 0.05

^**^P < 0.01

### Correlations between career adaptability and psychological capital and turnover intention

Pearson correlation analysis indicated that turnover intention had low negative correlations [47] with career adaptability and psychological capital (*r* = -0.176, *P* < 0.01; *r* = -0.203, *P* < 0.01). The career adaptability (*r* = 0.556, *P* < 0.01) and its four dimensions had moderate positive correlations with psychological capital (*P* < 0.01). (Table 2).

### Multiple linear regression analysis for the relationship between career adaptability and turnover intention

Table 3 displayed the results of multiple linear regression analysis to identify influencing factors of turnover intention. In the regression model, the results indicated that years of work were independent predictors of turnover intention, especially for more than 5 years and less than or equal to 10 years (*β* = 0.171, *P* = 0.014). In addition, career adaptability was significantly negatively associated with turnover intention after controlling for demographic factors (*β* = -0.169, *P* < 0.01). The statistically significant regression equation was revealed (*F* = 3.906, *P* < 0.001), which explained 8.7% of the variance in turnover intention.

**Table 3:** Multiple linear regression analysis for the relationship between career adaptability and turnover intention (*N* = 276)

Variables	*B*	*SE*	*β*	*t*	*p*
Constant	12.768	1.467	—	8.705	< 0.001
Years of work(≤ 1)					
> 1 and ≤ 3	0.011	0.536	0.001	0.020	0.984
> 3 and ≤ 5	0.812	0.739	0.071	1.099	0.273
> 5 and ≤ 10	1.410	0.569	0.171	2.481	0.014
> 10	-0.326	0.560	-0.040	-0.582	0.561
Professional qualification certificate	0.735	0.424	0.120	1.735	0.084
Monthly income (≤ 3000)					
3001–4000	0.539	0.501	0.087	1.077	0.282
4001–5000	0.793	0.543	0.111	1.461	0.145
> 5000	-1.003	0.705	-0.109	-1.424	0.156
The number of residents cared for per day	-0.494	0.579	-0.068	-0.854	0.394
Career adaptability	-0.035	0.012	-0.169	-2.695	0.007

*R*^*2*^ = 0.117, *AdjR*^*2*^ = 0.087, *F* = 3.906, *P* < 0.001, *B* = unstandardized regression coefficient, *SE* = standard error *β* = standardized regression coefficients

### The mediating effect of psychological capital between career adaptability and turnover intention

**Fig. 2:**
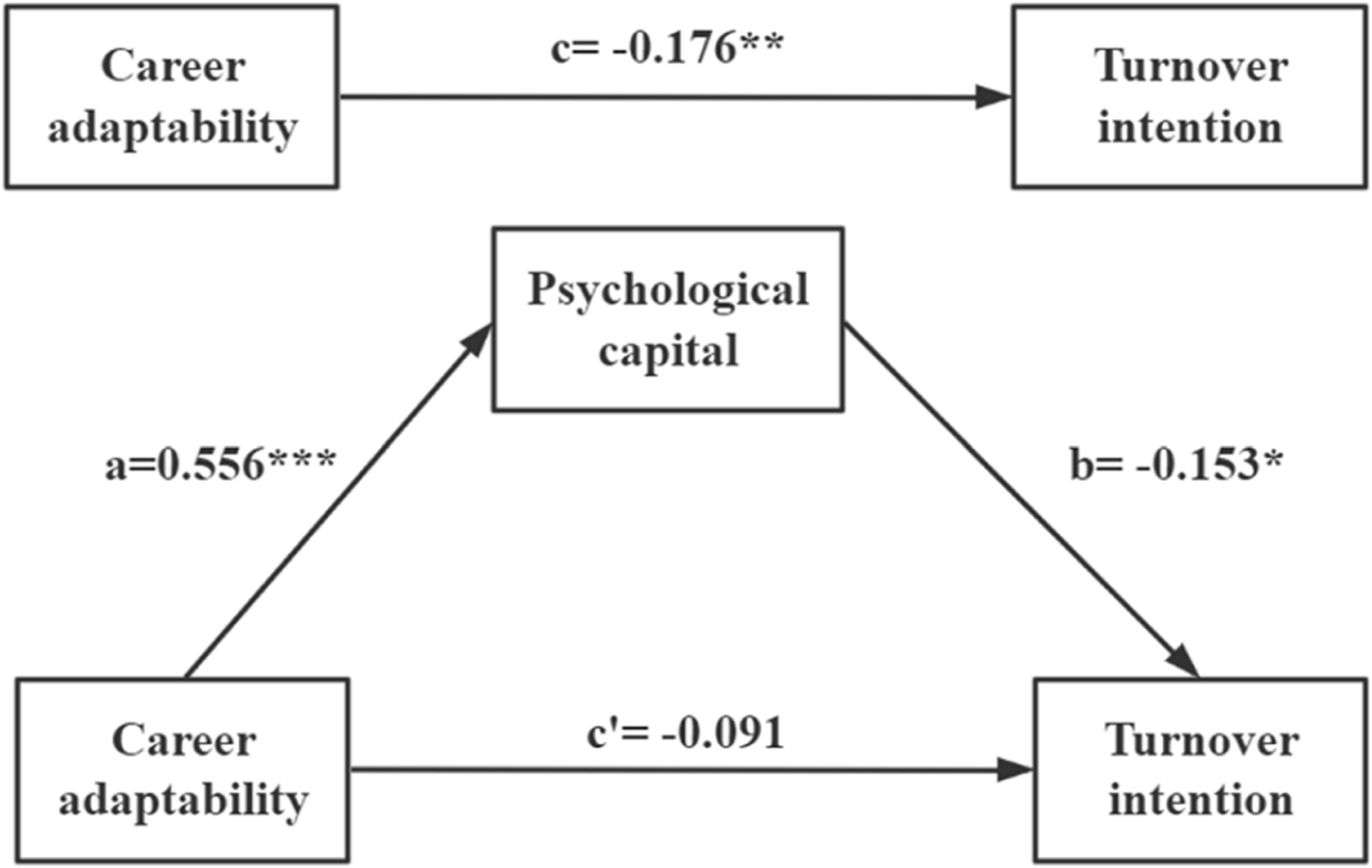
Psychological capital mediated the relationship between career adaptability and turnover intention. Note: *P < 0.05, **P < 0.01, *** *P* < 0.001

Figure 2 visually displayed the results of our theoretical model. Career adaptability had a significant positive impact on psychological capital (*β* = 0.556, *P* < 0.001). Psychological capital had a significant negative impact on turnover intention (*β* = -0.153, *P* < 0.05). However, career adaptability had no direct effect on turnover intention (*β* = -0.091, *P* > 0.05). A total significant negative impact of career adaptability on turnover intention was discovered (*β* = -0.176, *P* < 0.01). Thus, psychological capital played a complete mediating role in the relationship between career adaptability and turnover intention. As shown in Table 4, the mediating effect of psychological capital was -0.085, accounting for 48.3% of the total effect.

**Table 4:** Testing the mediating effects of psychological capital between career adaptability and turnover intention

Path	Effect	*β*	*SE*	Bootstrap95%CI (bias-corrected)	*p*	a*b/c
CAAS → PCS → TIS	Total effect	-0.176	0.012	-0.059 ~ -0.011	0.006	48.3%
	Indirect effect	-0.085	0.008	-0.032 ~ -0.002	0.025	
	Direct effect	-0.091	0.015	-0.046 ~ 0.011	0.219	
Concern → PCS → TIS	Total effect	-0.110	0.040	-0.152 ~ 0.008	0.077	NA
	Indirect effect	-0.079	0.020	-0.096 ~ -0.019	0.002	
	Direct effect	-0.032	0.042	-0.096 ~ 0.071	0.658	
Control → PCS → TIS	Total effect	-0.183	0.044	-0.217 ~ -0.048	0.002	42.6%
	Indirect effect	-0.078	0.027	-0.109 ~ -0.006	0.029	
	Direct effect	-0.104	0.052	-0.178 ~ 0.023	0.131	
Curiosity → PCS → TIS	Total effect	-0.144	0.044	-0.193 ~ -0.019	0.015	61.8%
	Indirect effect	-0.089	0.026	-0.119 ~ -0.018	0.007	
	Direct effect	-0.055	0.051	-0.138 ~ 0.062	0.428	
Confidence → PCS → TIS	Total effect	-0.194	0.043	-0.225 ~ -0.057	0.001	38.7%
	Indirect effect	-0.075	0.028	-0.110 ~ 0.000	0.049	
	Direct effect	-0.119	0.052	-0.187 ~ 0.016	0.098	

*TIS* Turnover intention scale, *CAAS* Career adapt-abilities scale, *PCS* Psychological capital scale, *β*, standardized regression coefficients, *SE* Standard error, *CI* Confidence interval, *NA* Not applicable

**Fig. 3:**
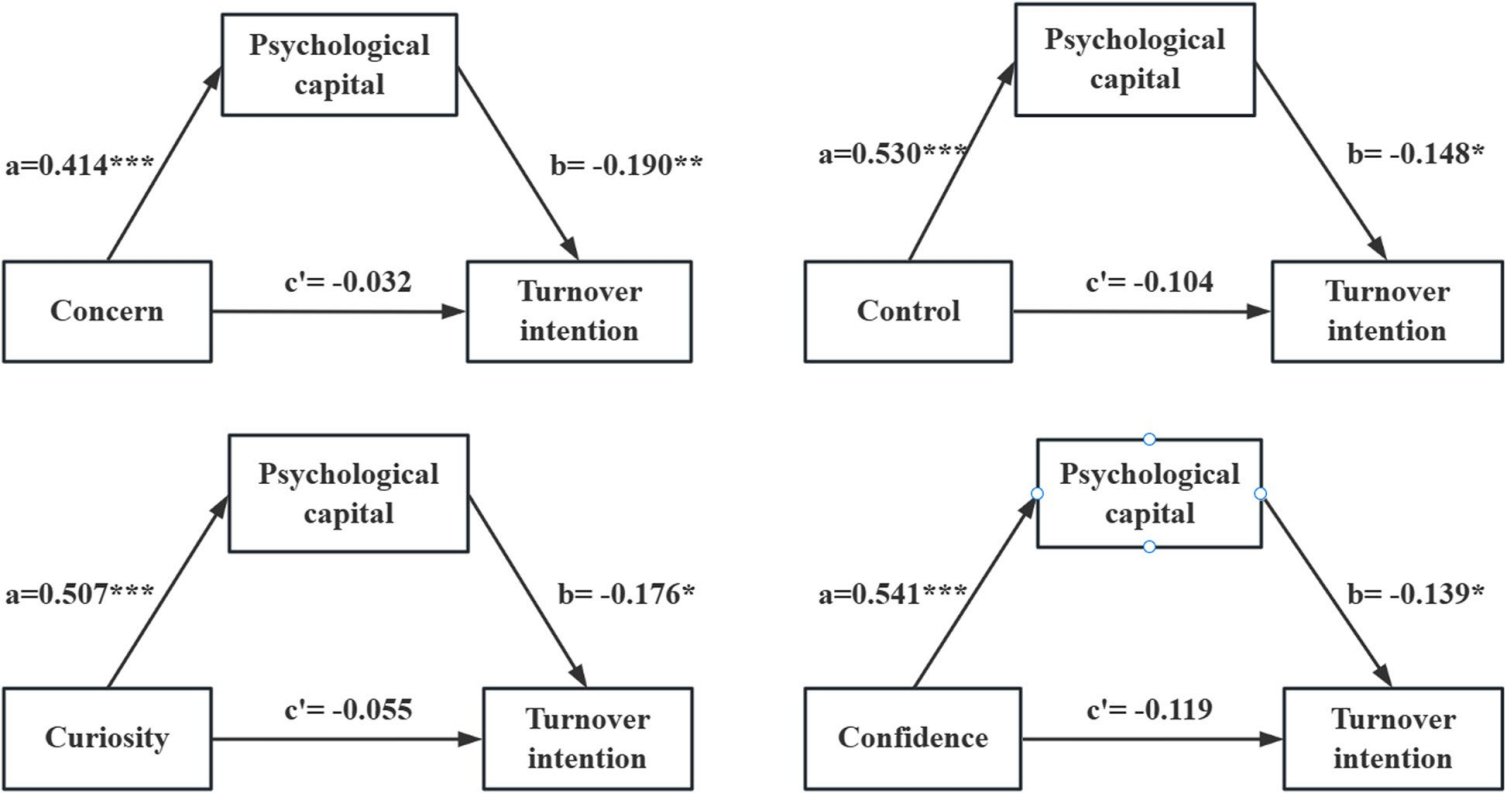
Psychological capital mediated the relationship of concern, control, curiosity, and confidence with turnover intention. Note: *P < 0.05, **P < 0.01, *** *P* < 0.001

To further understand the specific mediating effects, four models were constructed to examine how psychological capital mediated the relationship between the dimensions of career adaptability and turnover intention. The results indicated that psychological capital mediated the association between control(*β* = -0.078, *P* < 0.05), curiosity(*β* = -0.089, *P* < 0.05), confidence(*β* = -0.075, *P* < 0.05) dimensions and turnover intention. According to the proportion of indirect effect divided by total effect (a*b/c), the largest indirect effect was generated through curiosity (61.8%). (Fig. 3, Table4).

## Discussion

This study sought to investigate the relationship between career adaptability and turnover intention among NAs in mainland China, and to determine whether psychological capital mediated the association between them. The findings of this study provide useful insights into reducing turnover intention of NAs. First, we identified that years of work were an independent predictor of turnover intention. Second, the results supported that career adaptability was significantly positively related to psychological capital and negatively related to turnover intention. Finally and most importantly, psychological capital mediated the association between career adaptability, especially the curiosity dimension and turnover intention, which clarified the specific mechanism from a psychological perspective and facilitated a scientific basis for further interventions.

It was found that NAs with 5 to 10 years of work had the highest level of turnover intention, consistent with earlier studies [48, 49]. This maybe because new staff tend to be full of enthusiasm and expectation for their work because they’ve just joined the organization. Those who have worked for more than 10 years usually own reasonable wages and have mastered the personality characteristics of residents; therefore, they are satisfied with their treatment and have established stable emotional bonding with the residents, which reduces their intention to leave. Consequently, managers of LTC facilities should pay more attention to this group who have worked for 5 to 10 years, and retain them by raising their salaries and welfare, and promoting good relationships between them and residents.

Career adaptability is identified as an important determinant in reducing turnover intention. The results showed that career adaptability was significantly negatively associated with turnover intention. Thus, hypothesis1 was confirmed and this finding was compliant with previous studies in other occupations [16, 27]. As career construction theory illustrates [26], career adaptability can enable employees to adjust to changing career environment and job requirements through their concern, control, curiosity, and confidence. During their career progress, NAs are more likely to stay within the organization when they are focused on the future orientation and career vision, proactive in implementing career plans, willing to explore their unknown career development, and confident in their abilities to solve professional problems. Conversely, upon facing career challenges, those with low career adaptability lack these abilities to cope with difficulties and have trouble in regaining a dynamic balance between individual and organization, which trigger their intention to leave voluntarily. In summary, the findings re-emphasized the importance of assessing and intervening in career adaptability which played as a modifiable predictor of NAs’ turnover intention.

The mediation analysis suggested that psychological capital played a complete mediating role in the relationship between career adaptability and turnover intention. Specifically, higher levels of career adaptability were associated with better psychological capital (path *a)*, which in turn led to less turnover intention (path *b*). Hence, hypotheses 2 and 3 were confirmed. Yet the result could not support that there existed a direct effect of career adaptability on turnover intention (path *c’*). In other words, NAs with higher career adaptability are less likely to have intentions to leave owing to their better psychological capital. On the one hand, it has been shown that good career prospects are positively related to employees' job expectations and satisfaction, and facilitate good adjustments to their job roles [31]. Once NAs achieve external individual-organization balances and receive positive feedback on their work, further they will enhance internal psychological capital. They tend to be hopeful about their work, confident about their nursing abilities, and don't give up easily when they encounter hardships, so they do not consider quitting easily. On the other hand, studies have reported that psychological capital has a positive impact on organizational commitment [36, 50]. Employees with better psychological capital tend to keep a strong sense of belonging to their jobs, remain in close contact with colleagues, and are more willing to stay in the organization and play their roles [18, 40, 41]. NAs need to maintain good communication with their leaders and colleagues, and actively participate in organizational activities to promote an enjoyable team climate and a sense of belonging to their jobs. Furthermore, we found that psychological capital mainly mediated between curiosity and turnover intention through four additional mediation models. Curiosity represents "what I want to do in the future" and reflects a strong tendency to acquire knowledge and explore career prospects; meanwhile, it has a significant positive effect on hope and resilience [37]. Hence, this dimension could be highlighted to enhance psychological capital in future career planning. In summary, we can consider introducing the overall improvement of career adaptability and psychological capital into interventions to reduce turnover intention in the future.

Career adaptability is considered as the attitudes, competences, and behaviors making an individual better suited for the job [29]. First, it is recommended that government and nursing home managers provide NAs with more opportunities for advancement and occupational skill level recognition to stimulate job expectations, increase curiosity to explore their roles, and maintain high psychological capital. Second, psychological education courses should be included in the occupational training process to develop appropriate understandings of their work, and build a sense of professional responsibility and self-worth. Last, certified NAs in the US are required to attend continuing education courses annually to maintain their certifications [51]. It is suggested that nursing home managers regularly conduct professional knowledge and skills training for NAs to enhance their caregiving abilities, and promote adaptability and psychological capital during their career development.

In response to the personnel shortage, the General Office of the State Council's opinion on promoting the development of senior care stated that vocational colleges and universities were encouraged to establish senior care majors and cultivate more NAs students [52]. It is worth investigating whether highly-educated NAs have high levels of career adaptability and, if necessary, measures should be formulated for them. An online career adaptability improvement program was developed for college graduates [53], which consisted of three components: knowledge and awareness of self and work environment, self-directed coping related to career behaviors, and environmental interactions with career decision-making and adjustment. We can consider developing online career planning courses to promote NAs students’ greater career adaptability and less turnover intention, and facilitate career transitions in the future.

### Implications for practices and limitations

Inspired by the findings in this study, the government and management of nursing homes are encouraged to pay attention to career adaptability of NAs. Since NAs with middle school education and long working years are the primary and experienced staff in LTC facilities, it is recommended that administrators develop appropriate programs based on the mechanism of this study to reduce turnover intention and maintain workforce stability. Moreover, given reducing turnover intention of highly educated NA students is conducive to improving the overall quality of aged care. Before they formally enter the job, career planning courses are suggested to provide for them in schools to improve career adaptability, increase psychological capital, and reduce their turnover intention in the future.

Nevertheless, a few limitations should be noted. First, the generalizability of findings was limited because the data were collected from one city through convenience sample sampling. Future studies are recommended to expand the sample to further clarify the mediating association among the variables. Second, some participants with low education levels may have difficulty understanding the contents of questionnaire. During the COVID-19 epidemic, the researcher had to use online questionnaires rather than explaining the contents to participants via a face-to-face method, which may affect the results of this study.

## Conclusions

The management of LTC facilities should focus on assessing the level of career adaptability of NAs. Psychological capital mediated the relationship between career adaptability and turnover intention. The overall improvement of career adaptability and psychological capital is conducive in reducing turnover intention. Targeted interventions are recommended to improve career adaptability and reduce turnover intentions by increasing career curiosity, thereby maintaining the sustainable development of the LTC workforce. Moreover, online career adaptability programs can be developed for NA students to improve their psychological capital and facilitate career transitions.

## Data Availability

The datasets analyzed during the current study are available from the corresponding author on reasonable request.

## Abbreviations

LTC: Long-term care
NA: Nursing assistant
TIS: Turnover intention scale
PCS: Psychological capital scale
CAAS: Career adapt-abilities scale
